# Ten years of marine evolutionary biology—Challenges and achievements of a multidisciplinary research initiative

**DOI:** 10.1111/eva.13389

**Published:** 2023-01-07

**Authors:** Kerstin Johannesson, Erica H. Leder, Carl André, Sam Dupont, Susanne P. Eriksson, Karin Harding, Jonathan N. Havenhand, Marlene Jahnke, Per R. Jonsson, Charlotta Kvarnemo, Henrik Pavia, Marina Rafajlović, Eva Marie Rödström, Michael Thorndyke, Anders Blomberg

**Affiliations:** ^1^ Tjärnö Marine Laboratory, Department of Marine Sciences University of Gothenburg Strömstad Sweden; ^2^ Natural History Museum University of Oslo Oslo Norway; ^3^ Department of Biology and Environmental Science University of Gothenburg, Kristineberg Marine Research Station Fiskebäckskil Sweden; ^4^ International Atomic Energy Agency Principality of Monaco Monaco; ^5^ Department of Biology and Environmental Science University of Gothenburg Gothenburg Sweden; ^6^ Department of Marine Sciences University of Gothenburg Gothenburg Sweden; ^7^ Department of Genomics Research in Ecology & Evolution in Nature (GREEN) Groningen Institute for Evolutionary Life Sciences (GELIFES) De Rijksuniversiteit Groningen Groningen The Netherlands; ^8^ Department of Chemistry and Molecular Biology University of Gothenburg Gothenburg Sweden

**Keywords:** Baltic Sea, perspective, reference genomes, research centre

## Abstract

The Centre for Marine Evolutionary Biology (CeMEB) at the University of Gothenburg, Sweden, was established in 2008 through a 10‐year research grant of 8.7 m€ to a team of senior researchers. Today, CeMEB members have contributed >500 scientific publications, 30 PhD theses and have organised 75 meetings and courses, including 18 three‐day meetings and four conferences. What are the footprints of CeMEB, and how will the centre continue to play a national and international role as an important node of marine evolutionary research? In this perspective article, we first look back over the 10 years of CeMEB activities and briefly survey some of the many achievements of CeMEB. We furthermore compare the initial goals, as formulated in the grant application, with what has been achieved, and discuss challenges and milestones along the way. Finally, we bring forward some general lessons that can be learnt from a research funding of this type, and we also look ahead, discussing how CeMEB’s achievements and lessons can be used as a springboard to the future of marine evolutionary biology.

## 1 THE BIRTH AND ORGANIZATION OF CeMEB

Toxic chemicals, acid rain and ozone depletion were early eye‐openers for global scale anthropogenic impacts threatening ecological processes, but it was not until the start of the new millennium that scientists began to raise serious concerns about the evolutionary effects of global scale threats (Palumbi, 2001). For example, in marine ecosystems, it became obvious that effects of over‐exploitation, habitat fragmentation, warming and ocean acidification are major drivers of evolutionary change and that both common and rare species are at risk of being negatively impacted. In fact, populations and species unable to adapt to the new regimes, or unable to migrate to more suitable geographical areas, will likely go extinct. To find out more about the potential for species and populations to adapt, an increased research focus on the lowest level of biodiversity, the genetic level, was needed.

Partly inspired by the European Union FP6‐SUSTDEV project on high‐throughput genomics (Marine Genomics 2004–2008), a group of marine researchers in 2007 jointly felt that evolutionary consequences of climate change, including impact on the intraspecific level, were not being appropriately addressed in ongoing marine research projects. In addition, marine species were poorly represented in evolutionary research generally. Thus, we decided to build a new transdisciplinary programme focused on marine evolutionary biology and apply to a call for long‐term (10 year) funding of "Linnaeus Centres of Excellence" launched by the Swedish research councils (VR and Formas). We built the application around eight target species representing a broad range of marine taxa that have important roles in coastal marine ecosystems. The overarching aim was to perform fundamental and innovative research, taking calculated risks while having high expectations. Our application—"*Adaptation to Changing Marine Environments*"—was funded in July 2008, and this was the start of the Centre for Marine Evolutionary Biology (CeMEB). The main, long‐term objectives of CeMEB were bold and summarised as five questions:

- To what extent have organisms evolved following recent large‐scale and rapid environmental changes?
- What are the potentials for evolutionary change in key marine species?
- Which mechanisms at molecular and organismal level drive rapid adaptation?
- What is the role of plasticity in the evolution of new adaptations?
- How frequently and why will populations and species go extinct under different scenarios of future environmental change?

The new Centre rested on three main pillars: First was ready access to a large‐scale and recently established (*ca*. 8000 year old) environmental shift from oceanic conditions to nearly freshwater in the Baltic Sea (Snoeijs‐Leijonmalm et al., 2017) generating a salinity gradient, partly very steep, along the Swedish coast. Over this gradient, many marine species exhibit rapid genetic transitions (Johannesson & André, 2006), and this setting offers a potential for both experimental and comparative analyses of evolutionary processes underlying local adaptation and acclimatization (Johannesson et al., 2020). The second pillar was the next‐generation sequencing (NGS) methods that allowed for the development of new genomic resources for non‐model organisms (only a handful of marine species had a published reference genome at that time). The third pillar was the potential of a transdisciplinary Centre of Excellence to combine various disciplinary skills to build stronger competences and to attack new questions, including combining empirical data with mathematical modelling in order to hindcast species’ histories from genetic data and forecast species’ survival and distribution under new environmental conditions.

Starting with 10 senior scientists CeMEB grew rapidly through the hiring of outstanding graduate students, postdocs and young researchers. Additional faculty and young scientists were attracted to the activities at the Centre and participated as members on their own funding (Table 1). Our ambitions were to build an open and sharing environment which valued all members (independent of age, gender, cultural background etc.) equally. A signature activity in developing the CeMEB's research profile and activities were the biannual assemblies that included all members and to which we invited experts from other countries or disciplines. These (in total) 18 assemblies became a key platform for networking through presenting ongoing research, discussing preliminary results, making plans for new research collaborations, and not least for exciting scientific discussions. Members of our Advisory Board were also invited to our meetings and took an active part in many discussions and presentations.

**TABLE 1:** CeMEB in brief: key deliverables, funding and gender balance during the funding period

Indicator	2008–2018
CeMEB Assemblies	18
Workshops and courses	57
International Conferences	2 (4)
Members	89
Ratio female/male	51/49
Number of PhD theses	24 (30)
Number of journal publications	529
Linnaeus funding	8.7 m€
University funding	1.5 m€
Additional external funding	30 m€

Note

Figures in brackets indicate deliverables completed 1–2 years after termination of the 10 years of funding but initiated during the funding period.

**FIGURE 1:**
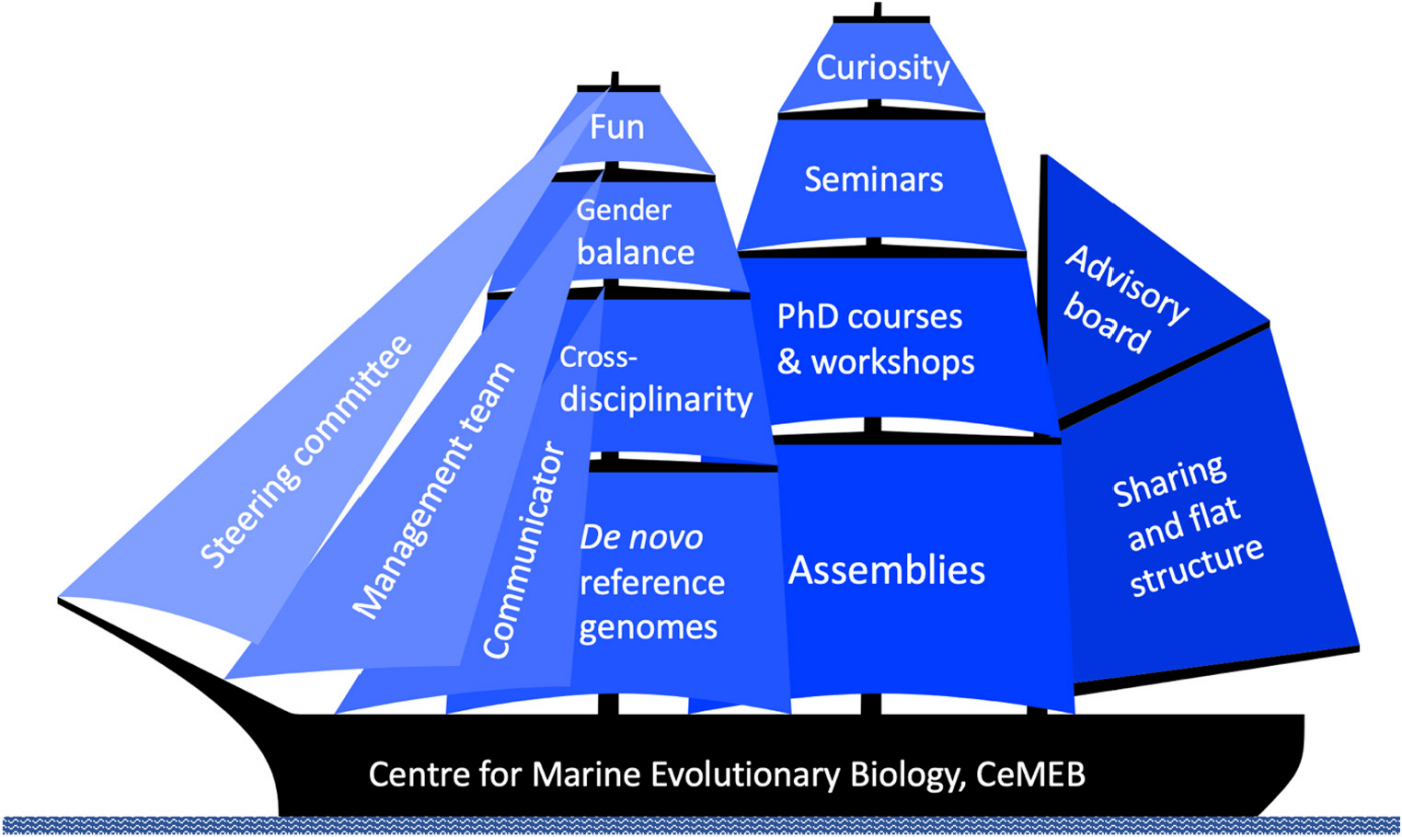
The organization of CeMEB illustrated as a sailing ship, where the different sails illustrate the most important driving forces of the centre's scientific progress

The assemblies were kept informal with short presentations and plenty of time set aside for discussion. The success of the assemblies was evidenced by >80% of members attending the meetings, on average. While the assemblies were the main activity of CeMEB, progress depended on a multitude of other components, such as, the development of draft genomes for seven marine species, important scientific input and influences of advisory board and assembly guest researchers, and not least, open and inspiring discussions involving everyone. Literary, CeMEB acted as a large sailing ship, for which progress was due to several different sails that were able to catch winds (research ideas) generated both inside and outside CeMEB (Figure 1).

**FIGURE 2:**
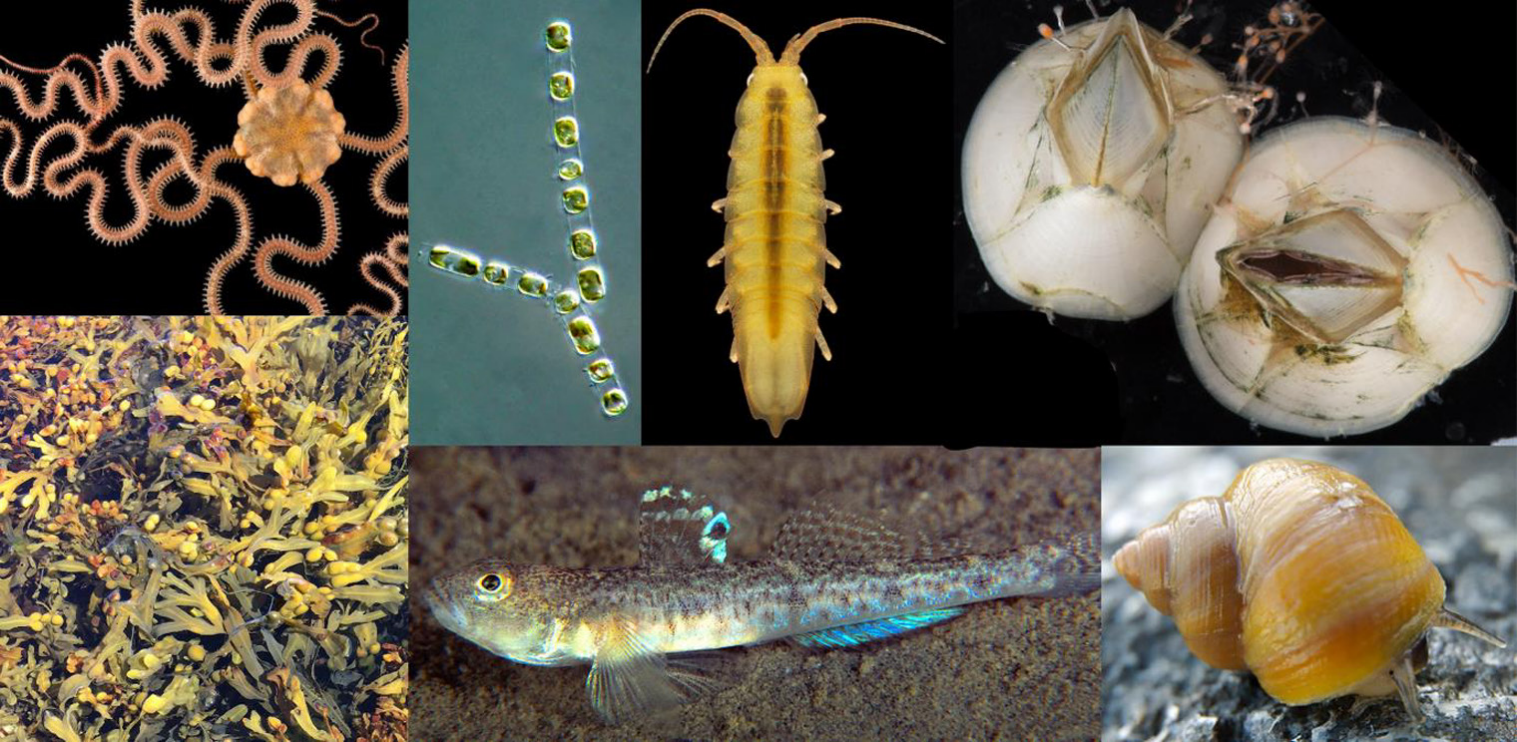
The seven CeMEB target species for which draft reference genomes have been developed. From upper left: the brittlestar *Amphiura filiformis*, the diatom *Skeletonema marinoi*, the isopod *Idotea balthica*, the barnacle *Balanus improvisus*, the bladderwrack *Fucus vesiculosus*, the sandgoby *Pomatoschistus minutus* and the snail *Littorina saxatilis*

As CeMEB started to address the main objectives it became obvious that much initial groundwork would be required to build a range of genomic resources for non‐model organisms. To start up parallel sequencing projects for seven marine species to provide *de novo* reference genomes was a bold proposition in 2010, not least as several of them represented species with both large and very heterogeneous genomes. By developing reference genomes for our target species, we aimed to make progress beyond earlier achievements that had used standard molecular tools for that time, such as mtDNA and microsatellite markers. However, the large size and high variability of the genomes (such as a high content [>40%] of genomic repeats, and extremely high genetic diversity with ~5% nucleotide diversity in coding regions of the barnacle *Balanus* (*Amphibalanus*) *improvisus*; Alm Rosenblad et al., 2021), meant that our ambitious aim to establish seven new genomes from different major groups of organisms (Figure 2) became a substantial challenge. Following the first Illumina‐based reference genomes, we have continued to improve several of the reference genomes by long read (PacBio) sequencing with the invaluable help of many collaborators.

## 2 CeMEB SCIENTIFIC HIGHLIGHTS

### 2.1 Local adaptation—ecological angles

Research within CeMEB has not only been broad with respect to marine taxonomy, but also with respect to topics. Understanding the impact of ocean acidification has been an urgent issue from the start. Ocean acidification is an important driver of directional selection and organisms’ capacities to tolerate and/or adapt will be critical to long‐term survival. Ocean acidification has already led to an ~30% increase in the average acidity of the ocean and could lead to a doubling in acidity by the end of the century. Before CeMEB, the established paradigm predicted that ocean acidification would drive negative effects primarily on marine calcifiers, as the change in seawater chemistry would compromise their ability to make or maintain shells and skeletons. However, early contradictory results showed that the response of calcifiers to ocean acidification was unexpectedly diverse. Some species were negatively impacted while others showed no or even positive responses to low pH (Eriander et al., 2015; Havenhand & Schlegel, 2009; reviewed by Wittmann & Pörtner, 2013). Investigating these contradictions was critical since development and implementation of solutions relies on our ability to project future impacts on marine species and ecosystems (Dupont et al., 2021; Dupont & Pörtner, 2013).

In CeMEB the impact of ocean acidification was investigated in a wide range of organisms from phytoplankton to fish. This research has in the first place contributed to the revision of the initial paradigm. Furthermore, it has dissected the mechanisms underlying organism responses and highlighted the key roles of acid‐base regulation (Stumpp et al., 2012), energy acquisition (Pansch et al., 2014), and feeding physiology (Stumpp et al., 2013). Different physiological (Dorey et al., 2013; Ventura et al., 2016) and evolutionary (De Wit et al., 2016; Sunday et al., 2014; Thor & Dupont, 2015) responses were observed when organisms were exposed to conditions within or outside their present ranges of variability. This led to the idea that local adaptation to extremes of present variability was the key to predict species and population sensitivity to ocean acidification (Vargas et al., 2017, 2022).

Investigating the impact of salinity on marine organisms’ ability to adapt locally has also been an issue of high relevance to CeMEB, not least since reduced salinity is a likely outcome of continued climate change in the Baltic Sea region. Examples of successful adaptations down to extremely low salinities (<5‰) include bladder wrack *Fucus vesiculosus*, a foundation species on rocky shores, and the isopod *Idotea baltica*, a main grazer of fucoid seaweeds. In the Baltic Sea, an exceptionally high density of this grazing isopod has resulted in the evolution of much higher constitutive levels of chemical defences in *Fucus*, compared to populations at higher salinities at the Swedish west‐coast (Nylund et al., 2012). Experiments under a future ocean acidification‐scenario revealed that both chemical defence and tissue strength are impaired in *Fucus* and, as a consequence, the seaweed becomes more susceptible to grazing and mechanical stress (Kinnby, Toth, et al., 2021; Kinnby, White, et al., 2021). Species distribution modelling suggests that the future range shift of *Fucus* in the Baltic area will primarily be determined by a predicted reduction in salinity, causing a dramatic shrinkage of its distribution range (Jonsson et al., 2018). The loss of *Fucus* as a habitat and food source for *Idotea* will add to the direct negative effects of a changing abiotic environment on the future distribution range of the isopod (Kotta et al., 2019). The association between *Fucus* and *Idotea* in the Baltic Sea offers a promising model system for experimental and theoretical studies of plant‐herbivore interactions under climate change, as illustrated by this CeMEB research. To further improve our ability to understand and model this system, more experimentally derived data are required on local adaption in physiological tolerance limits (Kinnby et al., 2020; Wood et al., 2016) and direct interactions between the two species. The integration of such ecological data will enable more powerful predictive modelling of the future distribution and abundance of the species and highlight the key role of evolution in shaping present and future sensitivity to changing physical conditions in coastal waters (Calosi et al., 2016).

CeMEB research has also demonstrated selection and local adaptation of behavioural and reproductive traits along the Baltic salinity gradient. Prior to CeMEB, studies of sand goby (*Pomatoschistus minutus*) had demonstrated indirect salinity effects on sexual selection via nest site availability (empty mussel shells; Forsgren et al., 1996; Jones et al., 2001; Singer et al., 2006). Research in CeMEB built on these foundations by investigating *direct* effects of salinity on reproduction within the wide range of salinities (3–35 PSU) inhabited by the species. Since the sand goby is an externally fertilizing fish, local adaptation of gamete function in relation to salinity is particularly interesting. *De novo* sequencing of the sand goby genome together with common‐garden experiments with sperm assays and cross‐population matings in different salinities, allowed a team of CeMEB researchers to explore the importance of local adaptation of gametes along the species’ geographical distribution (Leder et al., 2021; Lindström et al., 2021; Svensson et al., 2017). This research also resulted in a new concept, ‘immigrant reproductive dysfunction’, as an important mechanism for reduced gene flow, local adaptation and speciation (Svensson et al., 2017). Investigations of a related but invasive species (round goby, *Neogobius melanostomus*) focusing both on the genomic characterization (Adrian‐Kalchhauser et al., 2020) and on adaptation to increased salinities during its ongoing invasion of brackish and marine habitats, offered unique possibilities of studying local adaptation in real time (Green et al., 2019, 2021).

### 2.2 Local adaptation—genetic angles

The euryhaline barnacle *Balanus improvisus* is widespread along the Swedish coast‐line, exhibits extreme tolerance to very low salinities (<1 PSU; Sundell et al., 2019), and is a truly brackish water species with optimal growth at low/intermediate salinities (Wrange et al., 2014). This species was brought into CeMEB as a tractable model for studies of the evolution of osmoregulatory mechanisms in marine invertebrates, but little genetic/molecular information was available before the start of CeMEB, except for studies of rRNA genes and their phylogeny. CeMEB research has extended the *B*. *improvisus* gene‐list by sequencing and characterizing several genes, such as the octopamine receptors involved in the detection of antifouling substances (Lind et al., 2010), the α‐subunit of the Na^+^/K^+^ ATPase transporter (Lind et al., 2013), the full repertoire of eight aquaporin genes (Lind et al., 2017), the rich repertoire of waterborne pheromone genes (Abramova, Lind, et al., 2019) and the sensory receptor genes expressed in the cyprid antennules (Abramova, Alm Rosenblad, et al., 2019). The analysis of aquaporin functionality has lately been extended by heterologous expression and characterization in yeast, showing that a subset of the barnacle aquaporins have hydrogen peroxide transport capacity, that is they act as peroxiporins (U. Lind, pers. commun.). This functional information provides important clues to the differential roles of aquaporins in osmoregulation and in how some aquaporins might participate in larval settling mechanisms where the curing of the cement involves hydrogen peroxide. Further analyses of these genes in other species may reveal additional important mechanisms for salinity adaptation since an aquaporin gene (Aquaporin 4) has been linked to an outlier SNP in salinity adaptation in sand goby (Leder et al., 2021).

How local adaptation over environmental gradients can be achieved in the face of gene flow is a major question in evolutionary biology. Ecotype formation in the snail *Littorina saxatilis* illustrates this issue well, for example, early studies involving single genes showed that steep genetic clines are formed across small‐scale environmental gradients (Johannesson et al., 1995). The first draft genome of *L*. *saxatilis* combined with a linkage map generated from a 200 offspring large *L*. *saxatilis* family became key tools for CeMEB researchers to perform detailed analyses of phenotypic and genomic variation across repeated ecotype contact zones. These investigations led to the first discoveries of the role of chromosomal inversions in local adaptation and ecotype formation (Faria, Chaube, et al., 2019; Westram et al., 2018). Later quantitative analyses of hybrid crosses to identify genomic regions involved in adaptive traits (Quantitative Trait Loci; QTLs) showed that many of the QTLs reside inside some of the large inversions (Koch et al., 2021). Inversions and other structural arrangements have recently become a major focus in studies of polymorphic and subdivided species (Merot et al., 2020; Wellenreuther & Bernatchez, 2018). In the snail, the role of these inversions is visible at both local and regional scales with strong correlations between environmental gradients and inversion frequencies (Westram et al., 2021). Notably, specific inversions contribute to adaptation over different, and uncorrelated, environmental axes and so allow for independent adaptation of different traits along each axis (Morales et al., 2019). Overall, inversions provide major barriers to gene flow in adaptive traits and can strongly support local adaptation and incipient speciation (Kirkpatrick & Barton, 2006), and this is indeed the case in the snails *L*. *saxatilis* (Westram et al., 2018) and the closely related *L*. *fabalis* (see Le Moan, et al., 2022). At the same time, other barriers, such as assortative mating and intrinsic genetic incompatibilities seem to play minor roles (Johannesson et al., 2020; Perini et al., 2020). Future studies will trace the inversion history and establish which loci inside the inversions drive adaptation, as well as investigate how the regulatory landscape is impacted by these inversions. A conceptually interesting research question is also the role of selection in explaining the high levels of heterogeneity found in wild populations. A suggestion emerging from CeMEB research is that divergent and balancing selection have key roles (Faria et al., 2019; Johannesson & Butlin, 2017; Nunez et al., 2021), but more work on this topic is urgently needed.

### 2.3 Modelling marine species distributions and genetic structures

Modelling is an important tool for understanding and predicting the frequency, and potential causes, of population and species extinction under different scenarios of future environmental change. CeMEB researchers have applied population modelling, for example, to predict population responses and investigate extinction risks of marine mammals along the Swedish coasts. Grey seal, harbour seal, ringed seal and harbour porpoise all have different spatial distributions, different life histories, and are affected by different environmental drivers. By linking a climatological forecast model to a stochastic population viability (PVA) model for the ringed seal, we found that this species will respond negatively to even modestly warmer average winter temperatures within the nearest 50 years. Availability of high‐quality sea ice, required for successful breeding, will limit the population size below historical carrying capacity (Sundqvist et al., 2012). The Baltic grey seal, on the other hand, has a more flexible life history and can breed on land or ice, but critically depends on the availability of energy‐rich herring and sprat during the autumn (Kauhala et al., 2017). CeMEB researchers developed a detailed dynamic energy budget model and demonstrated how deteriorating energy content in prey not only suppresses reproduction in the following year, but can cause long‐lasting inter‐generational effects in terms of retarded maternal body growth, delayed maturity and lower pup survival (Silva et al., 2020). The harbour seal has the most flexible life history and pups can swim at birth if needed. The Swedish population of harbour seals has increased in numbers after a near collapse 50 years ago. At present they are exposed to several pressures, such as food shortage due to overfishing, sporadic viral epidemics and hunting (Harding et al., 2018). Research within CeMEB led to a multi‐stressor PVA for a range of scenarios and the discovery that some combinations of environmental factors and hunting can lead to rapid collapse of this seemingly stable population (Silva et al., 2021).

Population projection models tailored to the life histories of different species are important tools in predicting responses to future environmental changes in different seas and for predicting shifts in spatial distribution. The models will lead to improved population viability analysis, for example in pinnipeds, taking maternal effects into account and will help guide management decisions. A future target would be to study the genetic components of life histories and how PVA models can be developed to include the evolution of life histories under environmental change.

Integrating genetic data and biophysical modelling to infer future dispersal patterns and distributions has been a hot topic in marine population biology over the past decade. Indeed, the start of CeMEB coincided with the development of seascape genetics that combines information on genetic structure and spatial connectivity to understand how environmental parameters influence the extent of genetic variation within and among populations (Selkoe et al., 2016). In CeMEB we developed a platform for biophysical modelling of marine dispersal within the Baltic Sea‐North Sea transition, based on ocean circulation models and biological traits. In a recent review within CeMEB, it was concluded that biophysical models generally contribute to explaining population genetic patterns (Jahnke & Jonsson, 2022). A seascape genetic approach using biophysical modelling was successfully used to understand population structure of the bladder wrack *Fucus vesiculosus* (Pereyra et al., 2013), cod (Barth et al., 2017) and the isopod *Idotea balthica* (De Wit et al., 2020), indicating that oceanographic barriers can limit dispersal. The possibility of vertical barriers shaped by local oceanographic conditions (pycnoclines) was further illustrated by the gene flow barrier between shallow and deep populations of the tunicate *Ciona intestinalis* (Hudson et al., 2020; Johannesson et al., 2018). Biophysical modelling further suggests that dispersal may limit necessary gene flow to allow future range shifts of local adaptations in the rapidly changing environment of the Baltic Sea (Jonsson et al., 2018).

Forward‐in‐time, individual‐based, spatially‐explicit models can be used to understand species’ distributions and predict future distributions. Using this approach CeMEB researchers showed that the emergence of spatially dominant clones during range expansion does not require invoking selection for, or against, specific genotypes (Rafajlović et al., 2017). Instead, selectively neutral edge effects stemming from low population density at the expansion front and predominantly short‐range dispersal provide an advantage to clonal over sexual reproduction resulting in a clonal wave. This model could explain the distribution of large clones of an otherwise sexual species complex (*Fucus vesiculosus*/*F*. *radicans*) in the Baltic Sea (Pereyra et al., 2009). Furthermore, CeMEB research has shown that tighter linkage between adaptive loci (e.g. inside inversions) typically allows a population to occupy a greater range than expected under loose linkage and that sometimes tight linkage is necessary to avoid global extinction (Eriksson & Rafajlović, 2021). Occasional selfing can support a species to adapt over a wider range of environmental conditions than in obligate outcrossers, and also, plasticity in the adaptive trait may facilitate range expansion (Eriksson & Rafajlović, 2022). An important component here is to distinguish signatures of plasticity in the adaptive trait from putatively neutral plasticity in any fitness‐indicator trait that is not under direct selection. Using mathematical models, CeMEB researchers showed that making this distinction can be difficult based on reaction norms inferred from reciprocal‐transplant experiments, except for a limited number of special cases (Eriksson et al., 2022).

### 2.4 Marine genomics and management issues

While species distributions and ecology are widely assessed in monitoring of natural populations, genetic diversity of common species is rarely implemented in this type of survey (Brodersen & Seehausen, 2014). Evolutionary principles and genetic information can be invaluable in the conservation of species and populations, and the usefulness of genetic methods are rapidly expanding as new molecular technologies and statistical tools are developed (Allendorf et al., 2013). In CeMEB we developed genetic knowledge and tools to inform management in several commercially important fish species such as Atlantic cod (*Gadus morhua*) and wrasses (e.g. *Labrus bergylta*, *Ctenolabrus rupestris* and *Symphodus melops*, these latter used as cleaner fish to combat sea lice parasites in salmon aquaculture). For the cork‐winged wrasse (*Symphodus melops*), together with colleagues we established a reference genome (Mattingsdal et al., 2018) and detected a strong genetic break separating populations in the Skagerrak, where the wrasses were captured, from those in western Norway to which the fish were translocated (Mattingsdal et al., 2020). This strong genetic divergence enabled us to identify escapees from salmon farms as well as introgression into native populations (Faust et al., 2018, 2021). These findings have prompted explicit guidelines for the import and use of cleaner fish in Norwegian salmon aquaculture (Halvorsen, Skiftesvik, Durif, et al., 2021; Halvorsen et al., 2021). Genome‐wide genotyping and analysis of genome architecture of Atlantic cod uncovered cryptic population structure (Barth et al., 2017; Svedäng et al., 2019), and strong local adaptation in the Baltic Sea (Barth et al., 2019; Berg et al., 2015), which now forms the basis of stock separation in fisheries management (Hemmer‐Hansen et al., 2019). Similar evidence of strong local adaptation appears in many other commercial and/or foundation species (Han et al., 2020; Johannesson, Le Moan, et al., 2020; Knutsen et al., 2022; Le Moan et al., 2021), which challenges traditional management approaches. In the near future, we anticipate that extensive (in space and time) whole genome sequencing surveys (Barth et al., 2019; Han et al., 2020) and experimental studies corroborating functional significance of genomic outliers (Hill et al., 2019) will substantially improve our understanding of local adaptation and how locally adapted populations should be managed.

Studies in the late 1900s (reviewed in Boström et al., 2014) raised the awareness of eelgrass (*Zostera marina*) as a priority species in marine coastal conservation in the North Sea and Baltic Sea. However, at the start of CeMEB there was little knowledge on genetic diversity and structure of eelgrass in these seas. Recent progress has resulted in the first reference genome (Olsen et al., 2016), an improved version (Ma et al., 2021), transcriptomes (Franssen et al., 2011; Jueterbock et al., 2016), studies of methylation patterns (Jueterbock et al., 2019), and somatic mutations (Yu et al., 2020) that have collectively increased our understanding of the adaptive potential of the species. Eelgrass is also a priority species in conservation and ongoing work involves protection, restoration and monitoring. Recent protection strategies aim to incorporate an evolutionary dimension. Areas with high genetic diversity and high connectivity were identified by CeMEB researchers as priority areas (Jahnke et al., 2020) and have been communicated to regional authorities and will be included in current efforts to expand Natura 2000 areas. In terms of restoration, test planting has already been performed at candidate restoration sites which were suggested in Jahnke et al. (2020) as optimal for metapopulation connectivity based on oceanographic modelling and network theory. The technical developments and the combined efforts of research and management undertaken in CeMEB (e.g. through the two associated EU‐funded research programmes BaltGene and Bambi) have been highlighted by the European Commission as important for assessing the efficiency of the EU’s network of MPAs and defining management units (https://ec.europa.eu/environment/integration/research/newsalert/pdf/553na1_en‐eelgrass‐conservation‐insights.pdf) and have contributed to the Swedish Government now starting up a genetic monitoring programme covering key aquatic species of Swedish lakes and coasts, including, for example, large spatial‐scale genomic assessment of eelgrass, bladderwrack, cod and herring at regular temporal intervals (briefly described in Klütsch & Laikre, 2021).

## 3 OVERALL EXPERIENCES FROM THE CeMEB PROGRAMME

### 3.1 Did we reach our targets?

In the original application, we tried to look 10 years into the future and set realistic but ambitious (and partly high‐risk) goals for our research. Did we achieve the goals we formulated, and which milestones became critical during the development of the research to reach these goals? As we started CeMEB it became obvious that much groundwork was required in order to address many of our original research questions. A lot of necessary initial work was thus dedicated to the building of genomic resources. With progress in sequencing and annotation genomic resources were increasingly included in diverse studies of adaptations, evolutionary capacity and speciation. Many of the questions we raised in the original proposal still lack clear answers. Nevertheless, CeMEB has added much new data describing species’ evolution and adaptation across both shallow and steep environmental gradients: the former illustrated by the salinity gradient into the Baltic Sea while the latter exemplified with adaptation over within‐shore microenvironmental clines. These studies clearly illustrate potentials for rapid adaptation in some species, in particular when that adaptation is a replication of an adaptation to a similar condition elsewhere in the species’ distribution—i.e. parallel evolution (Butlin et al., 2014; Johannesson, Le Moan, et al., 2020; Morales et al., 2019). CeMEB studies have also shown that survival in new environments induces new costs to individuals (Kinnby et al., 2020; Nylund et al., 2012) and sometimes induces the establishment of new life‐history traits (Ardehed et al., 2015). Studies of the mechanisms involved in local adaptation have contributed new knowledge at both molecular and organismal level, such as the important role of chromosomal inversions for adaptation in some species. The role of plasticity in the establishment of range margins beyond which adaptation abruptly fails (despite plasticity) has been theoretically scrutinized only recently within modelling work in CeMEB (Eriksson & Rafajlović, 2022). We have also addressed the risk of extinction in various modelling approaches (Jonsson et al., 2018; Sundqvist et al., 2012), and modelling has been used to reach a better understanding of seemingly non‐adaptive life‐history strategies, such as the switch to widespread cloning in bladder wracks inside the Baltic Sea (Rafajlović et al., 2017).

One of the most important roles of CeMEB has been to establish resources that open up new ways to approach research questions in marine evolutionary biology. Moreover, many young researchers in (as well as outside) CeMEB have been inspired and trained and are now ready to take the next steps with challenging questions such as the mechanisms of plasticity, the role of genomic architecture (e.g. inversions), the maintenance of high genetic diversity, and how these features affect evolutionary processes. Finally, new tools are currently being developed (see below) that will take us even closer to the important goal of revealing the evolutionary capacity for adaptation to global change.

Although most research within CeMEB has involved organisms and conditions found in the Baltic Sea‐North Sea gradient, it can be argued that the impact goes well beyond this scale. Many studies have included general mechanisms and models that apply to many other systems worldwide, for example osmoregulation (Sundell et al., 2019), chromosome inversions (Westram et al., 2018), and effects of plasticity on range expansion (Eriksson & Rafajlović, 2021). In fact, the Baltic Sea has been highlighted as a time‐machine in terms of effects of global change and effective management (Reusch et al., 2018).

### 3.2 Some important lessons learned

It takes time to start up innovative science, as well as to initiate new collaborations among researchers from divergent disciplines. Without its long‐term funding, CeMEB would not have made the impact we see today. Although the annual funding CeMEB received from the research councils was not excessive—split across the ten co‐PIs it was just enough to hire a PhD student or Postdoc and pay running costs—it was the long‐term horizon (10 years) for developing new research that was most important, and which also facilitated additional external funding. Importantly, without that long funding period we would not have been able to develop novel genomic tools, and new, often transdisciplinary, collaborations. Here, the biannual CeMEB assemblies both inspired new research initiatives and provided networking opportunities that were especially important for younger members. Although the genome projects became much more challenging than we had imagined, they built excitement and enthusiasm within the group and raised a lot of interest and attention from researchers outside CeMEB, which also led to several new and important collaborations.

## 4 THE FUTURE OF MARINE EVOLUTIONARY BIOLOGY

Rapid advances in biotechnology provide many opportunities to increase the scope of marine evolutionary research from functional characterization and comparison of whole communities to detailed molecular biology and mechanistic insights within a species. Over the ten years of CeMEB, the power of sequencing technology has increased dramatically, facilitating the rapid accumulation of genetic and genomic resources for many marine species. However, despite notable studies (Alneberg et al., 2020; DeLong et al., 2006; Kashtan et al., 2014), we still need more information about very small taxa in the sea, such as protists, bacteria and viruses since they are essential for understanding the function and evolution of marine communities and ecosystems. In the last few years, several studies have applied metabarcoding and metagenomics approaches to characterize ecosystems (Bengtsson et al., 2017; Coutinho et al., 2018) and determine the effects of pollutants in marine systems (Pinnell & Turner, 2019), and researchers have even investigated communities using more functional approaches such as metatranscriptomics (Berg et al., 2018). Since so little is known about the marine taxa within many microbial groups these studies are just the beginning, but a classical approach using barcoding genes (e.g. a small portion of the 16S rRNA gene) is often not sufficient to identify microorganisms to the species level. Fortunately, molecular identification using longer amplicons or whole‐genome sequencing of microorganisms is today much more feasible through the use of unique molecular identifiers combined with long‐reads, (Karst et al., 2021) or single‐cell sequencing (Pachiadaki et al., 2019), and this allows for entire communities to be investigated from the smallest member.

At the other end of the scale, detailed investigation into the causative molecular mechanism for genetic variation is often desired, such as the genes that are the targets of selection. Although QTL mapping, genome‐wide association studies (GWAS), and outlier analyses can pinpoint the genomic regions that host such genes, or aid in estimating the number and effect of various loci, it is still quite difficult to find the causative SNP or mutation or even to link a mutation to a specific gene. This is because, in many cases, it is likely to be regulatory mutations that affect the phenotype (Wray, 2007), and regulatory sites can be quite distant from their target gene (Farley et al., 2015). Methods that allow for identifying regulatory elements have evolved in the past decade such that these studies are more feasible in non‐model organisms (Klemm et al., 2019). One particular method for this is ATACseq (Buenrostro et al., 2015). This method utilizes an engineered transposable element to insert sequencing adapters into open chromatin areas of DNA to amplify these segments, and these areas are then sequenced and mapped to the genome. Since open chromatin is an indicator of transcriptionally active areas of the genome, sequence peaks along the genome will highlight important regulatory areas in the tissue/condition of interest. When applied at the level of single cells, it can provide detailed information about the regulatory landscape for a phenotype of interest (Sinha et al., 2021). When applied at the level of populations, it may provide information on how the regulatory landscape can change due to environmental factors (de Carvalho Augusto et al., 2021) and thus provide insights into the adaptive versus plastic potential of populations.

Once a candidate gene is identified, further confirmation of the molecular mechanisms responsible for an adaptive trait will be needed, and CRISPR/Cas9 may be applied (Doudna & Charpentier, 2014). CRISPR/Cas9 techniques can be used to delete or insert sequences in the genome in order to knock‐out, knock‐down, or alter genes in a highly targeted manner. CRISPR/Cas9 has been applied in non‐model organisms to investigate shell coiling in snails (Abe & Kuroda, 2019), sex determination in Atlantic salmon (Wargelius et al., 2016) and various phenotypic traits in three‐spined stickleback (Wucherpfennig et al., 2019). From the genomic platforms now established for some of the CeMEB species, the leap is not too great to apply new approaches and technologies, such as CRISPR/Cas9. Whether we wish to study the evolution of species interactions or of molecular interactions, the next 10 years will be an exciting time for marine evolutionary biologists.

## CONFLICT OF INTEREST

We declare we have no competing interests.
